# Mitchell (Mitch) Mirkin (1962–2022) Acting Director, VA Research Communications

**DOI:** 10.1089/heq.2023.0008

**Published:** 2023-05-26

**Authors:** Erica Sprey

**Affiliations:** Office of Research and Development, US Department of Veterans Affairs, Baltimore, Maryland, USA.

**Figure:**
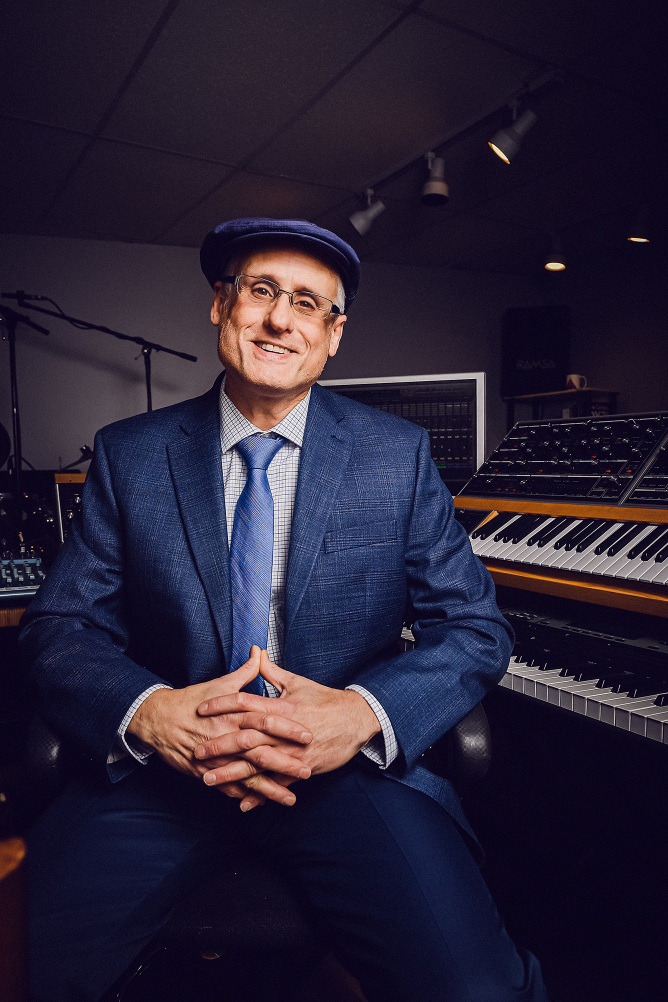
Photo by David Stuck, courtesy Baltimore Jewish Times

*A dedicated science communicator, Mirkin promoted the work of VA's Office of Research and Development and helped support Veterans' health and well-being.*

Mitch Mirkin, acting director of Communications for the Department of Veterans Affairs, Office of Research and Development, died suddenly at the age of 59, on March 18, 2022.

Mirkin spent more than 21 years working for VA Research Communications. He began as a writer, tasked with informing the scientific community, members of Congress, Veterans, and the general public about VA Research. Later, he was promoted to supervisory writer, where he managed a team of five communicators and advised senior leadership on the best ways to champion and promote VA Research.

“Mr. Mirkin had an outstanding ability to translate complex scientific findings into layman's terms,” said Dr. Carolyn Clancy, assistant undersecretary for Health for Discovery, Education and Affiliate Networks, Veterans Health Administration. “A highly impactful communicator, his relationship with content was more than transactional. He was extraordinarily attentive to the details of VA Research and deeply committed to demonstrating its outcomes.”

In 2020, when the COVID-19 pandemic first emerged, Mirkin was appointed acting director for VA Research Communications. In that position, he managed internal and external research communications that marshalled breaking information on SARS-CoV-2, potential treatments for COVID-19, and the development of new vaccines.

Mirkin was an expert resource for VA investigators and communicators. He was well known throughout the VA health care system and in research field offices across the United States. He also fostered cooperative relationships with external partners such as the Prostate Cancer Foundation.

“Mitch's service not only helped promote VA research achievements in the national consciousness, but also helped to facilitate advances in the health and care of our nation's Veterans. His absence leaves a tremendous void for VA Research, but his servant leadership on behalf of VA Research will never be forgotten,” said Patrick Helt, administrator, VA National Center for Rehabilitative Auditory Research in Portland, Oregon.

Mirkin was known for the breadth of his knowledge on scientific publishing, publication management, substantive editing, photojournalism, digital communications, and media relations. He was a talented writer and editor and employed a creative approach to storytelling—always looking for new and fresh ways to convey his message.

A native of Brooklyn, New York, Mirkin graduated cum laude from The City University of New York with a bachelor of arts degree in advertising and journalism. Later, he received a master of science degree in mass media arts and journalism from Clarion University in Western Pennsylvania. Before joining VA Research Communications, Mirkin worked as a publications manager for the Philadelphia Geriatric Center. He also taught English at Baltimore City Community College.

Outside of his professional life, Mirkin was an avid cyclist and belonged to The Baltimore Bicycling Club, often riding to his Baltimore office from his home in Pikesville, Maryland. He was a long-time member of the Randallstown Toastmasters club, writing for *Toastmasters Magazine* and mentoring others in public speaking at a Toastmasters club in Baltimore.

After turning 50, Mirkin rekindled an early interest in jazz music. He wrote and composed ∼40 pieces. Eventually, he formed his own band, *The Common Roots Jazz Ensemble*. The band recorded several jazz CDs, including “Dance of the DNA” and “Madison Avenue Shul.”

“When he spoke of his family, Mitch's love for his wife and sons came across so clearly. I also learned about his love of music and composition. Like Mitch, my late husband resumed his musical studies as an adult—his favorite musician was Cannonball Adderley. When I shared that with Mitch, he told me that it was Cannonball Adderley who got him interested in jazz as a youngster,” recalled Dr. Rachel Ramoni, VA's chief research and development officer.

Mirkin was married to Lauren (Jaffe) Mirkin. They had two sons, Naftali and Yehoshua (Shuey) Mirkin, and a daughter in-law, Carol.

“Mitch always had a clarity of purpose, thought and word that I admired. He brought a combination of professionalism, integrity and diplomacy to his work that few people possess; but along with those qualities he also brought genuine warmth, kindness and humility. All these things together made working with Mitch an absolute joy,” said Dr. Wendy Tenhula, deputy chief research and development officer.

